# Reduction of Duration of Antibiotic Therapy for Suspected Early-Onset Sepsis in Late-Preterm and Term Newborns After Implementation of a Procalcitonin-Guided Algorithm: A Population-Based Study in Central Switzerland

**DOI:** 10.3389/fped.2021.702133

**Published:** 2021-07-22

**Authors:** Jennifer Zihlmann-Ji, Christian Braun, Michael Buettcher, Markus Hodel, Dirk Lehnick, Martin Stocker

**Affiliations:** ^1^Department of Paediatrics, Neonatal and Paediatric Intensive Care Unit, Children's Hospital Lucerne, Lucerne, Switzerland; ^2^Department of Gynaecology and Obstetrics, Luzerner Kantonsspital, Lucerne, Switzerland; ^3^Department of Biostatistics, University of Lucerne, Lucerne, Switzerland

**Keywords:** early-onset sepsis, antibiotic stewardship, procalcitonin, neonates, biomarker

## Abstract

**Background:** Suspected early-onset sepsis (EOS) is the main reason for antibiotic therapy at the start of life. Prolonged antibiotic therapy for culture-negative sepsis is often reported. Antibiotic stewardship is mandatory due to the potential negative effects of unnecessary antibiotics. Procalcitonin (PCT)-guided therapy is one possible strategy with published evidence to shorten antibiotic therapy. The aim of this study is to analyze the feasibility and the performance of the published PCT-algorithm in the clinical setting without study support.

**Methods:** This is a retrospective, population-based study regarding duration of antibiotic therapy for suspected EOS in Central Switzerland between 2014 and 2018. All neonates >34 0/7 weeks of gestational age started on antibiotic therapy for suspected EOS within the first 3 calendar days of life were included. The Procalcitonin-guided algorithm according to the NeoPInS study was used as strategy to determine duration of antibiotic therapy.

**Results:** In a population-based cohort of 35,642 life born neonates, the duration of antibiotic therapy of 879 neonates (2.5% of the cohort) treated for suspected EOS was 4 calendar days (median, IQR 2–5). We observed a statistically significant reduction from 4 (median, IQR 3–6) to 3 calendar days (median, IQR 2–4) from 2014 to 2018. Duration of antibiotic therapy was independent of gestational age (late-preterm vs. term neonates), of the presence of risk factors or clinical signs, but dependent on the presence of abnormal laboratory measurements (C-reactive protein > 10 mg/l or leukocytopenia <5 Giga/l) before start of antibiotic therapy (*p* < 0.01).

**Conclusions:** PCT-guided therapy using the NeoPInS algorithm is feasible and may lead to reduced duration of antibiotic therapy for suspected EOS as reported in the original study. We observed a learning curve to the new algorithm which may be explained as change process. The use of biomarker to guide duration of antibiotic therapy for suspected EOS may have unintended consequences with prolongation of antibiotic therapy in some cases.

## Introduction

Internationally, length of exposure to antibiotics at the beginning of life for suspected neonatal early-onset sepsis (EOS) varies significantly (1, 2). Many publications are reporting high rates of antibiotic treatment for late-preterm and term neonates with only 0.01–0.5 out of 1,000 livebirths with blood culture positive EOS (3). The current management strategies are still less effective and antibiotic overtreatment at the beginning of life is a major problem (4–6). Unnecessary antibiotic exposure at the beginning of life may have negative effects on the development of a healthy bacterial microbiome and may induce antibiotic resistance (7, 8). Furthermore, connections have been made between antibiotic exposure after birth and chronic health care problems later in life (9). Whereas the start of antibiotic therapy for suspected EOS may be justified to lower mortality and morbidity in those cases of truly infected neonates, duration of therapy is often prolonged even in case of negative culture results (10). Fear of culture-negative sepsis is probably one of the main drivers for prolonged therapy (11).

Biomarker-guided therapy is one published approach reducing prolonged antibiotic therapy. The recently published NeoPInS study showed that duration of antibiotic therapy started for suspected EOS in late preterm and term neonates could be reduced with the use of a Procalcitonin (PCT)-guided algorithm (12). Duration of antibiotic therapy within this study was on average 55 h in the PCT-guided group of patients. Importantly, this study was powered to show the safety of the algorithm with an unchanged rate of reinfections and mortality. So far, the PCT-guided algorithm was not studied in other hospitals and circumstances. In addition, known as Hawthorne effect, there is a potential research participation effect leading to better results within a study than in reality (13).

At the Lucerne Children's Hospital, the PCT-guided decision-making algorithm for suspected neonatal EOS according to NeoPInS was introduced as standard of care in 2014. In this retrospective, population-based study we aim to analyze the impact of the implementation of PCT-guidance regarding duration of empirical antibiotic therapy for suspected EOS in late-preterm and term neonates of the area of central Switzerland from 2014 to 2018 and to show reproducibility of the results of the original study.

## Methods

This is a retrospective, population-based study in neonates regarding duration of antibiotic therapy for suspected EOS in Central Switzerland between 2014 and 2018 treated at Lucerne Children's Hospital. The study was approved by the regional Swiss ethical committee, which gave consent to collect individual data of all late preterm and term neonates started on antibiotic therapy within the first 3 days of life at the Children's Hospital of Lucerne between January 1st, 2014 and December 31th, 2018.

### Inclusion Criteria

All neonates >34 0/7 weeks of gestational age (GA) started on antibiotic therapy for suspected EOS within the first 3 days after delivery (birth = day 1) at Lucerne Children's Hospital between January 1st, 2014 until December 31th, 2018.

### Exclusion Criteria

Neonates started on antibiotic therapy for antibiotic prophylaxis (i.e., surgery, renal dysplasia) were excluded.

### Outcomes

The primary outcome is defined as duration of antibiotic therapy started for suspected EOS within the first 3 days of life, calculated annually and for 2014–2018. Secondary outcomes were duration of antibiotic therapy depending on GA (preterm infants 34 0/7–36 6/7 weeks of GA vs. term infants more than 36 6/7 weeks of GA), and on the presence of risk factors, and/or clinical signs, and/or laboratory signs possibly related to EOS. Therefore, the primary outcome is measured as change over time after the implementation of the NeoPInS algorithm with testing for possible confounding factors (secondary outcomes).

### Setting and Population

Lucerne Children's Hospital is a tertiary neonatal center and the only Children's hospital in Central Switzerland serving a population of ~806,000 people (Federal statistical office, bag.admin.ch 2017). Within that area, birth rates are on average around 7,000 deliveries annually. Deliveries (around 2,000 annually) are provided on the same campus as the Children's Hospital is located, in different regional hospitals (*n* = 9) or in delivery houses (*n* = 2). The standard care of neonates within the first few days of life is taken by the head of the obstetric departments of the different hospitals together with liaison pediatricians. All hospitals and delivery houses are part of a network for neonatal care of Central Switzerland. Lucerne Children's Hospital is the only neonatal center of this network and responsible for neonatal transports within the area and special care for all neonates, including intermediate and intensive care. Neonatal antibiotic treatment in Central Switzerland is only performed in our hospital.

According to the Swiss guidelines regarding management of neonates suspected for EOS, late preterm and term neonates with risk factors and without clinical signs for EOS were observed in each hospital at the delivery rooms or the mother and child unit (14). If clinical signs potentially related to EOS were observed, the physician on call for neonatal care at Lucerne Children's Hospital was contacted and the neonate was transferred. At Lucerne Children's Hospital care is provided by pediatric residents in close supervision of consultants for neonatology. All neonates stayed at the Children's Hospital until discharged at home.

### Strategies

Strategy regarding group B streptococci (GBS): Screening-based approach for GBS was in place in all hospitals requiring a vaginal and perianal GBS-swab (detecting method: PCR) after 35 weeks of gestation. In unknown or positive GBS-status antibiotics are given immediately after rupture of membrane or latest 4 h before delivery.

Strategy regarding start of antibiotic therapy: The Swiss guideline recommend to start antibiotic treatment (aminoglycoside combined with amoxicillin intravenously) for neonates with clinical signs possibly related to EOS (14). At absence of clinical signs of infection but exposed to risk factors, the neonate is clinically assessed every 4 h (signs of dyspnoea, temperature, peripheral circulation) for 48 h. In case of clinical signs possibly related to EOS, antibiotic therapy was immediately started. Before starting antibiotic therapy, blood cultures were taken.

Strategy regarding duration of antibiotic therapy: Procalcitonin-guided algorithm according to the NeoPInS study has been followed in each neonate started on antibiotic therapy due to suspected EOS (12). 12 and 24 h after starting empirical antibiotics PCT was measured. With both PCT values within the normal range [compare nomogram in the original study publication (12) and at our website www.nest-net.org], antibiotics were stopped 24 h after start. If PCT values were above the reference range, antibiotics were continued for at least 48 h. Continuation of antibiotic therapy after 48 h was dependent on culture results, clinical course, and C-reactive protein (CRP). In cases with high risk for culture negative sepsis antibiotics were continued for 5–7 days. Continuation of antibiotic therapy despite normal PCT values and negative cultures was possible and according to the assessment of the treating physician. The algorithm is available in the original study publication and at our research website www.nest-net.org (12).

### Definitions

The start of empirical antibiotics therapy for suspected EOS is defined as treatment within 3 calendar days after birth (birthday = day 1). Duration of antibiotics given are defined by calendar days. Every day with at least one dose of antibiotics given is counted. Risk factors for EOS were defined accordingly to the Swiss guidelines regarding management for suspected EOS: (i) Maternal group B streptococci (GBS) colonization (vaginal/ rectal swab: current or previous) (ii) prolonged rupture of membranes >18 h, (iii) chorioamnionitis (maternal fever >38°C plus two further symptoms: maternal leucocytosis, fetal tachycardia, painful or tender uterus, fetid amniotic fluid) (14). Clinical signs possibly related to EOS included tachypnoea, respiratory distress, apnoea, tachycardia/bradycardia, poor peripheral perfusion, mottling, temperature instability, lethargy, irritability, changes in tone, vomiting, poor feeding. Laboratory findings suggesting EOS included leukocytopenia (Leucocytes <5 Giga/l) or elevated C-reactive protein (>10 mg/l) prior to start of empirical antibiotics therapy (7).

### Data Collection

All neonates admitted to the children's Hospital of Lucerne within the first 3 days of life were identified through the electronic encoding data base of the hospital. Within that list, all neonates born in central Switzerland of at least 34 0/7 weeks of gestational age receiving empirical antibiotics for suspected early onset sepsis were identified by review of the electronical discharge letter. Risk factors, clinical signs, laboratory values, duration of antibiotic therapy and results of blood cultures and cerebro-spinal fluid cultures were manually collected from the electronical data set for all neonates fulfilling the inclusion criteria. The culture positive neonates were cross-checked with the electronical data base of positive cultures of the microbiology department. The number of deliveries within the catchment area were collected from each obstetric department as well as from the two delivery houses.

### Statistics

Descriptive data is presented as median and interquartiles [Q1, Q3] for continuous variables and as frequency (%) for categorical variables. Demographics and characteristics of patient groups were compared by using Fisher's exact test for categorical variables. Continuous variables were analyzed by Cuzick's non-parametric test for trend (a Wilcoxon-type test for trend), as well as by the Kruskal-Wallis test (15). Multiple ordered logistic regression analysis was performed to assess potential associations with duration of antibiotic therapy, applying model selection criteria such as Akaike's and Bayes' information criteria. Statistical analyses were performed using Stata (Version 15.1, StataCorp, College Station, Texas, USA).

## Results

The cohort contained 35,642 neonates of at least 34 0/7 weeks of gestational age, born from January 1, 2014 to December 31, 2018 in Central Switzerland. Admitted to the Children's Hospital of Lucerne within the first 3 days of life were 1988 neonates (5.6%) and 876 (2.5%) were started on antibiotic therapy due to suspected EOS (Table 1). There was a statistically significant trend for an increase of the proportion of hospitalized infants from 2014 to 2018 (from 4.6 to 5.9%; *p* < 0.001 according to Cuzick's non-parametric test for trend), as well as for the proportion of infants started on antibiotic therapy (from 2.2 to 2.6%; *p* = 0.024). The proportion of hospitalized infants started on antibiotic therapy remained unchanged between 44 and 46% (*p* = 0.629).

**Table 1:** Overview of analyzed population.

	**Live births**	**Hospitalized^**#**^**	**Antibiotic therapy^**#**^**	**Culture-proven early-onset Sepsis**
All infants	35,642	1,934 (5.4%)	876 (2.5%)	7 (0.02%)
Term infants	33,650	1,245 (3.7%)	635 (1.9%)	7 (0.02%)
Late-preterm infants	1,992	689 (34.6%)	241 (12.1%)	0
2014	6,904	317 (4.6%)	148 (2.2%)	2 (0.03%)
2015	6,926	361 (5.2%)	162 (2.3%)	2 (0.03%)
2016	7,142	387 (5.4%)	175 (2.5%)	2 (0.03%)
2017	7,251	431 (5.9%)	197 (2.7%)	1 (0.01%)
2018	7,419	438 (5.9%)	194 (2.6%)	0

# *Within the first 3 days of life*.

Most neonates (849 out of 876; 96.9%) started on antibiotics had clinical signs possibly related to EOS, whereas 385 (44.0%) had risk factors for EOS and 230 (26.3%) had laboratory abnormalities at start of antibiotic therapy (Table 2). Risk factors, clinical signs, and laboratory abnormalities possibly related to EOS were present in 122 neonates (13.9%). More than half of the neonates with antibiotic therapy (518 out of 876; 59.1%) were started on day 1, 286 (32.7%) on day 2 and 72 (8.2%) on day 3 of life. Annual cohorts had no significant differences within these baseline characteristics. Seven neonates had proven EOS with a positive blood culture. Characteristics of neonates with proven EOS are shown in Table 3. Seven out of 1988 hospitalized neonates died during hospitalization. None of them was sepsis related.

**Table 2:** Characteristics of infants with antibiotic therapy for suspected early-onset sepsis.

	**Antibiotic therapy^**#**^**	Risk factors^*^	Clinical signs^*^	Laboratory abnormalities^*^	Risk factors + clinical signs + laboratory abnormalities^*^
All infants	876	385 (44.0%)	849 (96.9%)	230 (26.3%)	122 (13.9%)
Term infants	635	280 (44.1%)	611 (96.2%)	218 (34.3%)	116 (18.3%)
Late-preterm infants	241	105 (43.6%)	238 (98.8%)	12 (5.0%)	6 (2.5%)
2014	148	55 (37.2%)	141 (95.3%)	42 (28.3%)	18 (12.2%)
2015	162	71 (43.8%)	153 (94.4%)	46 (28.4%)	20 (12.3%)
2016	175	77 (44.0%)	170 (97.1%)	46 (26.3%)	26 (14.6%)
2017	197	89 (45.2%)	192 (97.5%)	46 (23.4%)	29 (14.7%)
2018	194	93 (47.8%)	193 (99.5%)	50 (25.8%)	29 (14.9%)

# *Within the first 3 days of life;*

* *At start of antibiotic therapy*.

**Table 3:** Characteristics of neonates at start of antibiotic therapy with culture-proven early-onset sepsis.

**Case**	**Blood culture positive with**	Risk factors^*^	Clinical signs^*^	Laboratory abnormalities^*^	**Duration of antibiotic therapy**
1	GBS	GBS unknown	yes	no	14 days
2	GBS	no	yes	yes	15 days
3	Staph. epidermidis	no	Yes	yes	9 days
4	GBS	no	yes	yes	10 days
5	Staph. epidermidis	no	yes	yes	7 days
6	Staph. hominis	GBS unknown	yes	no	6 days
7	E. coli	no	yes	yes	10 days

* *At start of antibiotic therapy; Laboratory anomalies, CRP > 10 mg/l or Leucocytes <5 Giga/l; GBS, Group B Streptococci*.

**Figure 1:**
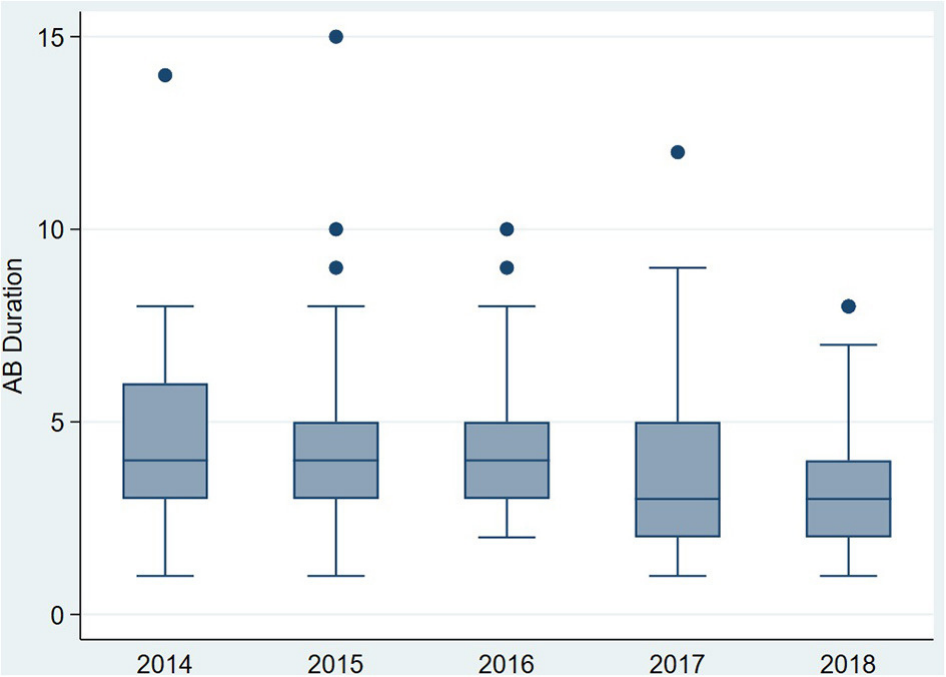
Primary outcome: duration of antibiotic therapy (median) for term and late-preterm infants above 34 0/7 weeks of gestational age from 2014 to 2018.

The duration of antibiotic treatment for all neonates was overall 4 calendar days (median, IQR 2–5). Analyses of annual duration of antibiotic therapy showed a reduction from 4 (median, IQR 3–6) to 3 calendar days (median, IQR 2–4) from 2014 to 2018 (Figure 1). The trend of annual reduction was significant analyzed by Cuzick's non-parametric test for trend (*p* < 0.001), as well as by the Kruskal-Wallis test (*p* < 0.001). In the multiple regression analysis, duration of antibiotic therapy was not depending on gestational age (late-preterm vs. term neonates) and clinical signs (since 96.9% of the patients had clinical signs, anyway), but on the presence of abnormal laboratory measurements (C-reactive protein > 10 mg/l or leukocytopenia <5 Giga/l) before start of antibiotic therapy (*p* < 0.001) and the calendar year of birth (*p* < 0.001) (Table 4A). Furthermore, the presence of risk factors is a useful variable in explaining the duration of antibiotic treatment (*p* = 0.15, but contributing to a favorable model selected using Akaike's and Bayes' information criteria, Table 4B). Abnormal laboratory measurements were associated with a prolongation of antibiotic therapy. The presence of risk factors in combination with abnormal laboratory measurements actually tended to lead into greater prolongation, whereas observed risk factors that were not confirmed by abnormal laboratory values resulted in potentially shorter duration of antibiotic treatment. A more recent year of birth was associated with a shortened duration of antibiotic therapy, which is best apparent using calendar year of birth as categorial variables (Tables 5A,B).

**Table 4A:** Ordered logistic regression: duration of antibiotic therapy by gestational age, calendar year of birth, risk factors, clinical signs, and laboratory abnormalities.

**Variable**	**Odds ratio**	**(95% CI)**	***p*-value**
Gestational age [week]	1.01	(0.96–1.07)	*p* = 0.68
Calendar year of birth [year]	0.81	(0.74–0.89)	*p* < 0.001
Risk factors [yes vs. no]^*^	0.84	(0.65–1.08)	*p* = 0.17
Clinical signs [yes vs. no]^*^	1.34	(0.59–3.06)	*p* = 0.48
Laboratory abnormalities [yes vs. no]^*^	2.41	(1.76–3.31)	*p* < 0.001

*Tables for (stepwise) ordered logistic regression – gestational age and calendar year of birth as continuous variables;*

* *at start of antibiotic therapy*.

**Table 4B:** Ordered logistic regression: duration of antibiotic therapy by calendar year of birth, risk factors, and laboratory abnormalities.

**Variable**	**Odds ratio**	**(95% CI)**	***p*-value**
Calendar year of birth [year]	0.81	(0.74–0.89)	*p* < 0.001
Risk factors [yes vs. no]^*^	0.83	(0.65–1.07)	*p* = 0.15
Laboratory abnormalities [yes vs. no]^*^	2.45	(1.84–3.27)	*p* < 0.001

*Proposed model based upon model selection utilizing Akaike's and Bayes' information criteria.*

* *At start of antibiotic therapy*.

**Table 5A:** Ordered logistic regression: duration of antibiotic therapy by gestational age, calendar year of birth, risk factors, clinical signs, and laboratory abnormalities.

**Variable**	**Odds ratio**	**(95% CI)**	***p*-value**
Gestational age [≥ week 37 vs. < week 37]	0.91	(0.68–1.22)	*p* = 0.54
Calendar year of birth [compared to 2014]			
2015	0.79	(0.52–1.21)	*p* = 0.28
2016	0.91	(0.60–1.38)	*p* = 0.66
2017	0.62	(0.41–0.93)	*p* = 0.022
2018	0.42	(0.28–0.62)	*p* < 0.001
Risk factors [yes vs. no]^*^	0.82	(0.63–1.05)	*p* = 0.12
Clinical signs [yes vs. no]^*^	1.32	(0.58–3.01)	*p* = 0.51
Laboratory abnormalities [yes vs. no]^*^	2.56	(1.88–3.49)	*p* < 0.001

*Tables for (stepwise) ordered logistic regression – gestational age and calendar year of birth as categorical variables;*

* *at start of antibiotic therapy*.

**Table 5B:** Ordered logistic regression: duration of antibiotic therapy by calendar year of birth, risk factors, and laboratory abnormalities.

**Variable**	**Odds ratio**	**(95% CI)**	***p*-value**
Calendar year of birth [compared to 2014]			
2015	0.79	(0.52–1.20)	*p* = 0.27
2016	0.91	(0.60–1.36)	*p* = 0.63
2017	0.62	(0.41–0.93)	*p* = 0.021
2018	0.42	(0.28–0.62)	*p* < 0.001
Risk factors [yes vs. no]^*^	0.82	(0.64–1.06)	*p* = 0.13
Laboratory abnormalities [yes vs. no]^*^	2.46	(1.84–3.28)	*p* < 0.001

*Proposed model based upon model selection utilizing Akaike's and Bayes' information criteria.*

* *At start of antibiotic therapy*.

## Discussion

Our retrospective, population-based study over 5 years showed an overall duration of antibiotic therapy started for suspected EOS of 4 calendar days (IQR 2–5). Interestingly, there was a continuous reduction from 2014 to 2018 from 4 calendar days in 2014 (IQR 3–6) to 3 in 2018 (IQR 2–4). Abnormal biomarker measurements (leukocytopenia below 5 g/l and/or CRP >10 mg/l) at time of suspicion of EOS independently has led to a longer duration of antibiotic therapy. In this era of globally increasing antibiotic resistance rates, the World Health Organization has highlighted the urgent need for enhanced antimicrobial stewardship programs. In addition, the importance of the individual microbiome and the development at the beginning of life makes antibiotic stewardship to a mandatory task for every physician caring for neonates.

Overall, 2.5% of our population in central Switzerland between 2014 and 2018 were treated with antibiotics due to suspected EOS within the first 3 days of life. This is comparable to other recently published population-based studies in Norway and the rate achieved by using the sepsis calculator (16, 17). A recent publication in California including 121 hospitals with a NICU reported a median of 7.3% of the live birth population exposed to antibiotics with a high variation from 1.6 to 42.5% (1). Our rate of culture-proven EOS was with 0.02% lower than internationally published but within the range published for a Swiss cohort between 2011 and 2015 (18). In our cohort, we didn't have any sepsis-related death which is in line with other recent publications showing a very low mortality for late-term and term neonates started on antibiotics due to suspected EOS (16).

There are not many studies published reporting duration of antibiotic therapy for suspected EOS in population-based cohorts. Comparing our results of duration of antibiotic therapy for suspected EOS with the literature, we conclude that the PCT-guided algorithm of NeoPInS may shorten antibiotic therapy overall. Fjalstad reported a duration of overall 6 days (median, IQR 5–7) for term neonates with culture-negative EOS and 4 days (median, IQR 3–5) for “rule-out” sepsis situations in a population-based cohort of 3,964 neonates treated with antibiotics for suspected EOS (16). Dretvic reported in a population from 3 Norwegian hospitals in a before-after setting with an educational intervention for a serial physical examination strategy to reduce antibiotic exposure an overall duration of antibiotic therapy of 108 h (median, IQR 60–144 h) before and 96 h (median, IQR 48–120 h) after the intervention for a cohort of late-preterm and term neonates (19). The main aim of this study was to reduce the percentage of neonates started on antibiotics, which was 2.5% in the pre-implementation phase, but below 2% in the post-implementation phase (1.8%). In a similar recent published population-based, single-center, quality improvement study, Vatne reported a duration of antibiotic therapy for term neonates suspected of EOS of 4.25 days (median, IQR 2–5) in the baseline phase and 5 days (median, IQR 2–5) in the intervention period (20). The percentage of neonates treated with antibiotics for suspected EOS was similar to our study in the baseline phase (2.9%), but significant lower in the intervention phase (1.3%). Within the NeoPinS study, we reported a duration of 55 h for the PCT-guided group (12). We are not able to compare our results directly with the NeoPins study due to the fact that we only captured calendar days with antibiotic treatment. Nevertheless, the overall duration of antibiotic therapy in our cohort and especially the duration at the beginning of the time frame was longer than reported in NeoPInS, whereas it came into the range of NeoPInS toward the end of the observation period. Our study may serve as proof of concept that the implementation of the NeoPInS algorithm is feasible and may lead to similar results in reality as in the study situation. One of the problems regarding the implementation of the NeoPInS algorithm is the time-dependent nomogram for PCT. Whereas, we implemented in our study the published nomogram into our standard procedure notes, the newly launched app for the NeoPInS algorithm may facilitate the implementation further.

The observed continuous reduction of duration of antibiotic therapy from 2014 to 2018 may be reasoned as learning curve to PCT guidance. Whereas, our center was part of the NeoPInS study, junior physicians and even consultants were not really familiar with the use of the algorithm due to regular rotations of the staff. Implementation of a new management algorithm means always a change. Not all staff members are convinced from the beginning and it takes time to adapt and to trust a new management strategy. Recent studies have shown that compliance with neonatal antimicrobial stewardship programs are not easy to obtain (21–23). On the other hand, implementing a new strategy means discussion and education about an important topic. We may conclude that implementation of a biomarker guided strategy needs some time to get its full potential impact and that educational interventions may have an additional effect. This is in line with the recent report of Dretvic using an educational intervention to successfully reduce exposure to antibiotics at the start of life (19).

Important to note, biomarker guidance may not have only beneficial effects. Abnormal biomarker measurements at the time of the first dose of antibiotics has led to a prolonged duration of antibiotic therapy. Even more, the effect of abnormal biomarker measurements showed the highest odds ratio for prolonged duration of antibiotic therapy in the multiple regression analysis. This shows the challenge of the use of biomarkerfor guidance of therapy: The heuristics of the human brain regarding decision making is potentially biased and works often in a dichotomized way (24). If a measurement is not normal (or negative), we have a tendency to interpret the result as abnormal or positive. Together with the fear of culture-negative sepsis, this may lead to even more prolonged antibiotic therapy. Therefore, a biomarker-guided management is not an easy-to-follow cook recipe without need of education and leadership. An educational empowering leadership style has shown to impact antibiotic stewardship programs and is a potential help to hinder unintended consequences (25).

The main weaknesses of our study are 2-fold: First, the low rate of culture proven EOS hinders us to make any conclusion regarding the safety of the NeoPInS approach in our study. Nevertheless, together with the original NeoPInS study, the data base is getting stronger showing that there are no missed sepsis cases and no sepsis-related mortality. Secondly, due to the implementation of the NeoPInS algorithm as the new standard for care, we did not have a control group. Therefore, it is not possible to make any judgment regarding the impact of the new strategy compared to the situation without algorithm. The significant trend of annual shortening of the duration of antibiotic treatment from 2014 to 2018 is a hint that there is a benefit through the new strategy, whereas we really do not know what had the biggest impact. The strengths of the study are the population-based cohort with an unchanged protocol and unchanged guidelines regarding management for suspected EOS within the 5-year time-frame.

We conclude, that PCT-guided therapy using the NeoPInS algorithm is feasible and may lead to reduced duration of antibiotic therapy for suspected EOS as reported in the original NeoPInS study. We observed a learning curve to the new algorithm which may be explained as change process. The use of biomarker to guide duration of antibiotic therapy for suspected EOS may have unintended consequences with prolongation of antibiotic therapy in some cases. Further studies analyzing an educational intervention and the impact of the newly launched NeoPInS app are potential next steps in the ongoing endeavor to improve antibiotic stewardship at the beginning of life.

## Data Availability Statement

The raw data supporting the conclusions of this article will be made available by the authors, without undue reservation.

## Ethics Statement

The studies involving human participants were reviewed and approved by Ethikkommission Nordwest und Zentralschweiz EKNZ. Written informed consent from the participants' legal guardian/next of kin was not required to participate in this study in accordance with the national legislation and the institutional requirements.

## Author Contributions

JZ-J, MS, and DL: study concept and design which was approved by all authors, supervision and monitoring data entry, and checking database for accuracy. JZ-J, MS, and CB: data collection. DL: statistical analysis. JZ-J, MS, DL, MB, and MH: analysis and interpretation of data. All authors read, critically revised and approved the manuscript, approved the final version, and agree to be accountable for all aspects of the work.

## Conflict of Interest

The authors declare that the research was conducted in the absence of any commercial or financial relationships that could be construed as a potential conflict of interest.

## References

[1] Schulman J Benitz WE Profit J Lee HC Dueñas G Bennett MV. Newborn antibiotic exposures and association with proven bloodstream infection. Pediatrics. (2019) 144:e20191105. 10.1542/peds.2019-1105 31641017

[2] Soll RF Edwards WH. Antibiotic use in neonatal intensive care. Pediatrics. (2015) 135:928–9. 10.1542/peds.2015-0707 25896842

[3] van Herk W Stocker M van Rossum AMC. Recognising early onset neonatal sepsis: an essential step in appropriate antimicrobial use. J Infect. (2016) 72:S77–82. 10.1016/j.jinf.2016.04.026 27222092

[4] Mukherjee A Davidson L Anguvaa L Duffy DA Kennea N NICE. neonatal early onset sepsis guidance: greater consistency, but more investigations, and greater length of stay. Arch Dis Child Fetal Neonatal Ed. (2015) 100:F248–9. 10.1136/archdischild-2014-306349 25079114

[5] Manzoni P Dall'Agnola A. Reducing unnecessary antibiotic exposure in preterm neonates: an achievable goal. Lancet Infect Dis. (2016) 16:1094–6. 10.1016/S1473-3099(16)30222-5 27452783

[6] Capin I Hinds A Vomero B Roth P Blau J. Are early-onset sepsis evaluations and empiric antibiotics mandatory for all neonates admitted with respiratory distress? Am J Perinatol. (2020). 10.1055/s-0040-1717070. [Epub ahead of print]. 32947642

[7] Schulfer A Blaser MJ. Risks of antibiotic exposures early in life on the developing microbiome. PLoS Pathog. (2015) 11:e1004903. 10.1371/journal.ppat.1004903 26135581 PMC4489621

[8] Fjalstad JW Esaiassen E Juvet LK van den Anker JN Klingenberg C. Antibiotic therapy in neonates and impact on gut microbiota and antibiotic resistance development: a systematic review. J Antimicrob Chemother. (2018) 73:569–80. 10.1093/jac/dkx426 29182785

[9] Stiemsma LT Michels KB. The role of the microbiome in the developmental origins of health and disease. Pediatrics. (2018) 141:e20172437. 10.1542/peds.2017-2437 29519955 PMC5869344

[10] Weiss SL Fitzgerald JC Balamuth F Alpern ER Lavelle J Chilutti M. Delayed antimicrobial therapy increases mortality and organ dysfunction duration in pediatric sepsis. Crit Care Med. (2014) 42:2409–17. 10.1097/CCM.0000000000000509 25148597 PMC4213742

[11] Klingenberg C Kornelisse RF Buonocore G Maier RF Stocker M. Culture-negative early-onset neonatal sepsis - at the crossroad between efficient sepsis care and antimicrobial stewardship. Front Pediatr. (2018) 6:285. 10.3389/fped.2018.00285 30356671 PMC6189301

[12] Stocker M van Herk W El Helou S Dutta S Fontana MS Schuerman FABA. Procalcitonin-guided decision making for duration of antibiotic therapy in neonates with suspected early-onset sepsis: a multicentre, randomised controlled trial (NeoPIns). Lancet Lond Engl. (2017) 390:871–81. 10.1016/S0140-6736(17)31444-7 28711318

[13] McCambridge J Witton J Elbourne DR. Systematic review of the Hawthorne effect: new concepts are needed to study research participation effects. J Clin Epidemiol. (2014) 67:267–77. 10.1016/j.jclinepi.2013.08.015 24275499 PMC3969247

[14] Stocker M Berger C McDougall J Giannoni E. Taskforce for the Swiss Society of Neonatology and the Paediatric Infectious Disease Group of Switzerland. Recommendations for term and late preterm infants at risk for perinatal bacterial infection. Swiss Med Wkly. (2013) 143:w13873. 10.4414/smw.2013.13873 24089151

[15] Cuzick JA. Wilcoxon-type test for trend. Stat Med. (1985) 4:87–90. 10.1002/sim.4780040112 3992076

[16] Fjalstad JW Stensvold HJ Bergseng H Simonsen GS Salvesen B Rønnestad AE. Early-onset sepsis and antibiotic exposure in term infants: a nationwide population-based study in Norway. Pediatr Infect Dis J. (2016) 35:1–6. 10.1097/INF.0000000000000906 26368059

[17] Achten NB Klingenberg C Benitz WE Stocker M Schlapbach LJ Giannoni E. Association of use of the neonatal early-onset sepsis calculator with reduction in antibiotic therapy and safety: a systematic review and meta-analysis. JAMA Pediatr. (2019) 173:1032–40. 10.1001/jamapediatrics.2019.2825 31479103 PMC6724419

[18] Giannoni E Agyeman PKA Stocker M Posfay-Barbe KM Heininger U Spycher BD. Neonatal sepsis of early onset, and hospital-acquired and community-acquired late onset: a prospective population-based cohort study. J Pediatr. (2018) 201:106–14. 10.1016/j.jpeds.2018.05.048 30054165

[19] Dretvik T Solevåg AL Finvåg A Størdal EH Størdal K Klingenberg C. Active antibiotic discontinuation in suspected but not confirmed early-onset neonatal sepsis—a quality improvement initiative. Acta Paediatr. (2020) 109:1125–30. 10.1111/apa.15202 31999863

[20] Vatne A Klingenberg C Øymar K Rønnestad AE Manzoni P Rettedal S. Reduced antibiotic exposure by serial physical examinations in term neonates at risk of early-onset sepsis. Pediatr Infect Dis J. (2020) 39:438–43. 10.1097/INF.0000000000002590 32301920

[21] Cantey JB Patel SJ. Antimicrobial stewardship in the NICU. Infect Dis Clin North Am. (2014) 28:247–61. 10.1016/j.idc.2014.01.005 24857391

[22] Patel SJ Rosen E Zaoutis T Prasad P Saiman L. Neonatologists' perceptions of antimicrobial resistance and stewardship in neonatal intensive care units. Infect Control Hosp Epidemiol. (2010) 31:1298–300. 10.1086/657334 20979494 PMC4526133

[23] Hersh AL Beekmann SE Polgreen PM Zaoutis TE Newland JG. Antimicrobial stewardship programs in pediatrics. Infect Control Hosp Epidemiol. (2009) 30:1211–7. 10.1086/648088 19852666

[24] Kahneman D. Thinking Fast and Slow. New York, NY: Farrar, Strauss and Giroux (2011).

[25] Steinmann KE Lehnick D Buettcher M Schwendener-Scholl K Daetwyler K Fontana M. Impact of empowering leadership on antimicrobial stewardship: a single center study in a neonatal and pediatric intensive care unit and a literature review. Front Pediatr. (2018) 6:294. 10.3389/fped.2018.00294 30370263 PMC6194187

